# Put on a Happy Face—It's a Lot Better Than Coumadin

**DOI:** 10.1161/JAHA.112.002444

**Published:** 2012-06-22

**Authors:** Rachel Lampert

**Affiliations:** Yale University School of MedicineNew Haven, CT

**Keywords:** atrial fibrillation, distress, happiness

Although medical factors predisposing to atrial fibrillation (AF) are well described, whether and how psychosocial factors influence the development of AF remains a nearly unexamined area. In this respect, AF lags far behind other cardiovascular conditions, such as coronary artery disease and ventricular arrhythmia, in which relationships between psychosocial factors and cardiac disease have been well established. Depression predicts the recurrence of ventricular arrhythmia in patients with implantable cardioverter-defibrillators over the long term,^1,2^ and negative emotions such as anger can acutely trigger ventricular arrhythmias in this population.^3^ Although the attempts of AF patients to identify psychosocial triggers for their episodes are familiar to all who participate in the care of these patients, to date, large systematic studies evaluating how emotion may affect AF are lacking. There is one small study of 54 AF patients undergoing cardioversion, in which 85% of those scoring high in depression, compared with 39% of those without depression, had a recurrence over the 2-month follow-up period.^4^ In the Framingham study, measures of anger, hostility, and tension predicted development of AF over 10 years in men but not in women.^5,6^

In this month's issue of the *Journal of the American Heart Association (JAHA),* Whang et al^7^ analyze data from the Women's Health Study of female health professionals to evaluate whether global distress, as measured by the Mental Health Index (a subset of the Short Form-36), and specific emotional components of distress predict development of AF. This is a large, prospective, well-known study, with long-term follow-up, a validated instrument for psychosocial evaluation, careful confirmation of the AF outcome, and comprehensive statistical analysis, including adjustment for multiple potential confounders at baseline as well as subsequent cardiac events. In the 30 746 middle-aged women studied, who were free of AF or other cardiovascular disease at baseline, 771 cases of AF occurred over the median 10-year follow-up period. There was no relationship between global distress or its components, including depression, and likelihood of development of AF.^7^ In a post hoc analysis of the individual components of the Mental Health Index, however, happiness was protective, lowering risk of AF by 30% for those who reported feeling happy some or most of the time compared to those who reported being rarely happy. It is heartening that only 4% of these female healthcare providers reported feeling happy just rarely, whereas 73% were happy most of the time.

Although it is post hoc, this finding is intriguing and warrants further study. The role of negative emotion in predisposing to coronary disease, ventricular arrhythmias, and other ills has been extensively described, with reports dating to the eighteenth century drawing a connection between anger and angina.^8^ The beneficial effects of positive affect, however, have just begun to be recognized. In laboratory studies of provoked mental stress, happiness attenuates stress-induced increases in fibrinogen in healthy individuals,^9^ and cardiovascular reactivity to stress is similarly decreased in those with a positive emotional style.^10^ In other short-term laboratory experiments, watching a comedy improved vascular function, as measured by brachial artery flow-mediated dilation and carotid arterial compliance. (Jerry Seinfeld, Ellen Degeneres, and Bill Cosby videos were included in that study).^11^ In daily life, happiness also alters physiological processes: Among healthy individuals, happiness is associated with lower daily heart rate and cortisol,^9^ and anxiety increases ambulatory diastolic blood pressure only in individuals experiencing low levels of happiness.^12^ A good mood also makes a difference clinically: Optimism decreases risk of cardiovascular death,^13^ and emotional vitality confers a decreased likelihood of developing coronary artery disease.^14^ How happiness exerts these beneficial effects is unknown. One postulated mechanism for the clinical beneficial effects of psychological well-being has been salubrious changes in health-related behaviors, such as smoking, alcohol intake, and exercise. However, the study by Whang et al^7^ controlled for these behavioral factors, which did not attenuate the impact of happiness, suggesting a direct and possibly autonomically mediated effect of positive affect. Determining the pathophysiological mechanisms by which happiness may decrease likelihood of AF is an important avenue of future research.

Ultimately, understanding psychosocial mechanisms of disease may lead to novel therapies. Interventions aimed at increasing positive affect, including those based on psychological principles as well as more complementary modalities such as meditation, have shown psychological effects (decreasing perceived stress) and also physiological effects in some disease states.^15^ Should the protective effects of happiness against AF be confirmed, examination of whether interventions that may increase positive affect could decrease risk of AF is another area for future investigation.

Also interesting is the primary finding, that global distress and depression were not associated with development of AF. This was somewhat surprising, given the prior findings linking depression and negative emotions to ventricular arrhythmias.^1–3^ The authors postulate that differences in autonomic influences on atrial versus ventricular arrhythmias may lie behind the lack of association between negative affect and AF; they note that AF is often vagally mediated, whereas negative emotions lower vagal activity. However, prior studies have described sympathetic influences on the atrium that are conducive to AF. Shortening of the atrial refractory period facilitates AF,^16^ and the shortest atrial refractory periods are seen in the morning, the time of the highest catecholamine levels.^17^ Direct sympathetic stimulation shortens the atrial refractory period as well.^18^ Furthermore, clinical use of sympathomimetic drugs such as dobutamine for stress echocardiography or inotropic support is well known to precipitate AF,^19^ and isoproterenol has been used in the past to aid induction of AF in ablation.^20^

It is possible that the method of initial identification of AF—self-report—could have confounded an association, if women who were more distressed or depressed were less likely to report AF. No data are available on the rate of “false negatives.” However, in a prior study of patients with AF, those with depression were more likely to report symptoms, not less likely,^21^ making this an improbable explanation.

A more probable explanation for the lack of association between negative emotional patterns and AF seen in the study by Whang et al^7^ may lie in the population studied: women. Prior studies have demonstrated an effect of anger, depression, and tension on future AF only in men, not in women.^5,6^ Whether negative emotion is associated with ventricular arrhythmias in women has not been adequately studied, as prior studies have not included sufficient women^3^ (a failing unfortunately not uncommon to studies in defibrillator populations). Nevertheless, ample evidence indicates that negative emotion has different physiological effects in women than in men. Hemodynamic and neuroendocrine response to laboratory stressors is greater in men than in women, although specific patterns of activation by sex vary according to the stressor.^22^ Women and men also show differences in brain activation in response to stress.^23^ Furthermore, estrogen attenuates tachycardia-induced atrial refractory period shortening,^24^ which could lead to differences between the sexes in propensity to AF with stress. Whether global distress and other negative emotions influence development of AF in men is another important avenue of further research that may be spurred by this thought-provoking study.
